# (*E*)-2-[4-(Diethyl­amino)­styr­yl]-1-methyl­pyridinium benzene­sulfonate mono­hydrate

**DOI:** 10.1107/S1600536811004156

**Published:** 2011-02-09

**Authors:** Hoong-Kun Fun, Narissara Kaewmanee, Kullapa Chanawanno, Suchada Chantrapromma

**Affiliations:** a X-ray Crystallography Unit, School of Physics, Universiti Sains Malaysia, 11800 USM, Penang, Malaysia; b Crystal Materials Research Unit, Department of Chemistry, Faculty of Science, Prince of Songkla University, Hat-Yai, Songkhla 90112, Thailand

## Abstract

The asymmetric unit of the title compound, C_18_H_23_N_2_^+^·C_6_H_5_O_3_S^−^·H_2_O, comprises a 2-[4-(diethyl­amino)­styr­yl]-1-methyl­pyridinium cation, a benzene­sulfonate anion and a solvent water mol­ecule. One ethyl substituent of the diethyl­amino group of the cation is disordered over two positions in a 0.73789 (9):0.26211 (9) ratio. The cation exists in the *E* configuration with respect to the C=C bond and the π-conjugated system is essentially planar with a dihedral angle of 0.82 (10)° between the pyridinium and benzene rings. The cation and anion are almost orthogonal with a dihedral angle of 86.71 (10)° between the π-conjugated system of the cation and benzene ring of the anion. In the crystal, mol­ecules are arranged into chains along [001] and adjacent chains are linked by weak C—H⋯O inter­actions. The crystal is further stablilized by O—H⋯O hydrogen bonds and weak C—H⋯π inter­actions.

## Related literature

For standard bond lengths, see Allen *et al.* (1987 ▶). For background to and applications of quaternary ammonium compounds, see: Chanawanno *et al.* (2010 ▶); Fun *et al.* (2010 ▶); Massi *et al.* (2003 ▶); Soprey & Maxcy (1968 ▶); Yabuhara *et al.* (2004 ▶). For related structures, see: Chanawanno *et al.* (2010 ▶); Kaewmanee *et al.* (2010 ▶).
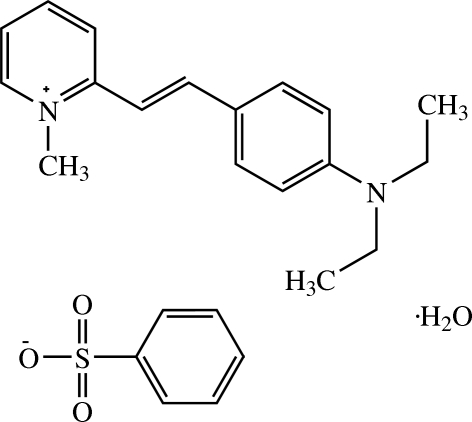


## Experimental

### 

#### Crystal data

C_18_H_23_N_2_^+^·C_6_H_5_O_3_S^−^·H_2_O
*M* *_r_* = 442.57
Monoclinic, 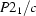

*a* = 9.9393 (5) Å
*b* = 17.9047 (9) Å
*c* = 13.2532 (7) Å
β = 100.715 (1)°
*V* = 2317.4 (2) Å^3^
*Z* = 4
Mo *K*α radiation
μ = 0.17 mm^−1^
*T* = 297 K
0.47 × 0.28 × 0.27 mm

#### Data collection

Bruker SMART APEXII CCD area-detector. diffractometer
Absorption correction: multi-scan (*SADABS*; Bruker, 2009 ▶) *T*_min_ = 0.924, *T*_max_ = 0.955
23078 measured reflections
6105 independent reflections
3770 reflections with *I* > 2σ(*I*)
*R*_int_ = 0.030

#### Refinement

*R*[*F* ^2^ > 2σ(*F* ^2^)] = 0.057
*wR*(*F* ^2^) = 0.173
*S* = 1.04
6105 reflections
293 parameters
H-atom parameters constrained
Δρ_max_ = 0.37 e Å^−3^
Δρ_min_ = −0.34 e Å^−3^

### 

Data collection: *APEX2* (Bruker, 2009 ▶); cell refinement: *SAINT* (Bruker, 2009 ▶); data reduction: *SAINT*; program(s) used to solve structure: *SHELXTL* (Sheldrick, 2008 ▶); program(s) used to refine structure: *SHELXTL*; molecular graphics: *SHELXTL*; software used to prepare material for publication: *SHELXTL* and *PLATON* (Spek, 2009 ▶).

## Supplementary Material

Crystal structure: contains datablocks global, I. DOI: 10.1107/S1600536811004156/sj5101sup1.cif

Structure factors: contains datablocks I. DOI: 10.1107/S1600536811004156/sj5101Isup2.hkl

Additional supplementary materials:  crystallographic information; 3D view; checkCIF report

## Figures and Tables

**Table 1:** Hydrogen-bond geometry (Å, °) *Cg*1 is the centroid of the C19–C24 ring.

*D*—H⋯*A*	*D*—H	H⋯*A*	*D*⋯*A*	*D*—H⋯*A*
O1*W*—H1*W*1⋯O2^i^	0.97	1.91	2.821 (3)	156
O1*W*—H2*W*1⋯O1^ii^	1.07	1.88	2.933 (3)	170
C1—H1*A*⋯O3^iii^	0.93	2.26	3.151 (3)	160
C3—H3*A*⋯O2^ii^	0.93	2.41	3.335 (4)	178
C4—H4*A*⋯O1*W*	0.93	2.50	3.338 (3)	149
C18—H18*B*⋯O3	0.96	2.45	3.371 (3)	162
C10—H10*A*⋯*Cg*1^i^	0.93	2.95	3.741 (2)	144

Symmetry codes: (i) 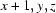; (ii) 
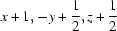
; (iii) 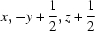.

**Fig. 1.**
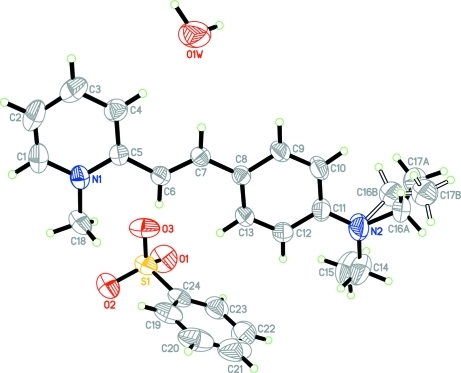
The asymmetric unit of (I) showing 40% probability displacement ellipsoids and the atom-numbering scheme. Atoms of the minor disorder component are linked by open bonds.

**Fig. 2.**
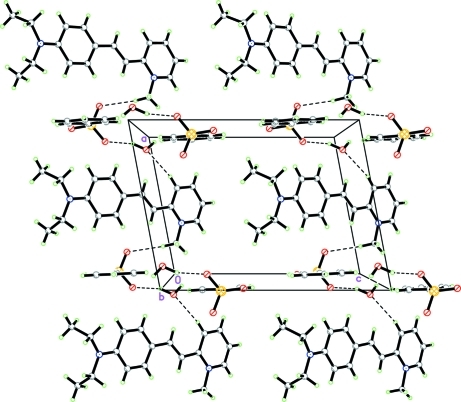
The crystal packing of the major component viewed along the b axis. The O—H···O hydrogen bonds and weak C—H···O interactions are drawn as dashed lines. Only the major component is shown.

## supplementary crystallographic information

### Comment

Quaternary ammonium compounds (QACs) are relatively low toxicity and wide-ranging antimicrobial agents that are commonly used for water treatment, food industry additives and hygienic care for both medical and domestic purposes (Yabuhara *et al.*, 2004). However, due to the long-term usage of common QACs such as benzalkonium chloride and cetylpridinium chloride, QAC resistant microorganisms have appeared. It was reported that some *Staphylococcus* spp. contain genes conveying resistance to this type of disinfectant (Massi *et al.*, 2003; Soprey *et al.*, 1968; Yabuhara *et al.*, 2004). Therefore, we have developed the novel pyridinium QACs which can overcome this *Staphylococcus*-resistant phenomenon by exhibiting strong anti-methicillin-resistant *Staphylococcus aureus* activity and reported this discovery in our previous work (Chanawanno *et al.*, 2010; Fun *et al.*, 2010). The title compound was the one among many pyridinium QACs which was synthesized in our laboratory hoping for a new antibacterial drug candidate. The antibacterial activity of this compound is under investigation and its crystal structure is reported here.

Fig. 1 shows the asymmetric unit of the title compound (I) which consists of the C_18_H_23_N_2_^+^ cation, C_6_H_5_O_3_S^-^ anion and one H_2_O molecule. The cation exists in the *E* configuration with respect to the C6═C7 double bond [1.337 (2) Å]. The π-conjugated system of cation (N1/C1–C13) is planar with an *r.m.s* deviation of 0.0215 (2) Å and the dihedral angle between the C1–C5/N1 pyridinium and the C8–C13 benzene rings is 0.82 (10)° with the torsion angle C5–C6–C7–C8 = -179.19 (17)°. One ethyl unit of the diethylamino moiety is disordered over two positions; the major component *A* and the minor component *B* (Fig. 1), with a refined site-occupancy ratio of 0.73789 (9)/0.26211 (9). The diethylamino group deviates from the attached C8–C13 ring and its conformation can be indicated by the torsion angles C11–N2–C14–C15 = 83.8 (4)°, C11–N2–C16–C17 = -95.3 (4)° for the major component *A* and 106.1 (7)° for the minor component *B*. The cation and anion are inclined to each other as indicated by the dihedral angle between the π-conjugated system of cation (N1/C1–C13) and the C19–C24 benzene ring of anion being 86.71 (10)°. The bond lengths (Allen *et al.*, 1987) and angles in (I) are in normal ranges and comparable with those for related structures (Chanawanno *et al.*, 2010; Kaewmanee *et al.*, 2010).

In the crystal packing, the cations, anions and water molecules are arranged into individual chains along the [001] direction (Fig. 2). The cations are linked to the anions and water molecules in neighboring chains by C—H···O weak interactions (Table 1 and Fig. 2) whereas the anions are linked to water molecule by O—H···O hydrogen bonds (Table 1). A C—H···π interaction involving the benzenesulfonate anion was observed (Table 1).

### Experimental

(*E*)-2-(4-(diethylamino)styryl)-1-methylpyridinium iodide (compound A, 0.14 g, 0.37 mmol) was prepared by a literature method (Kaewmanee *et al.*, 2010) and then was mixed with silver (I) benzenesulfonate (Chanawanno *et al.*, 2010) (0.10 g, 0.37 mmol) in methanol (100 ml). The mixture immediately yielded a grey precipitate of silver iodide. After stirring the mixture for 30 min, the precipitate of silver iodide was removed and the resulting solution was evaporated yielding the title compound as an orange solid. Orange block-shaped single crystals of the title compound suitable for *x*-ray structure determination was recrystallized from methanol by slow evaporation of the solvent at room temperature after a few weeks, Mp. 466–468 K.

### Refinement

All H atoms were placed in calculated positions to ride on their parent atoms, with d(O—H) = 0.97 and 1.07 Å, d(C—H) = 0.93 Å for aromatic and CH, 0.97 Å for CH_2_ and 0.96 Å for CH_3_ atoms. The *U*_iso_ values were constrained to be 1.5*U*_eq_ of the carrier atom for methyl H atoms and 1.2*U*_eq_ for the remaining H atoms. A rotating group model was used for the methyl groups. The highest residual electron density peak is located at 1.09 Å from H14B and the deepest hole is located at 0.72 Å from S1.

### Crystal data

**Table:**

C_18_H_23_N_2_^+^·C_6_H_5_O_3_S^−^·H_2_O	*F*(000) = 944
*M**_r_* = 442.57	*D*_x_ = 1.268 Mg m^−^^3^
Monoclinic, *P*2_1_/*c*	Melting point = 566–468 K
Hall symbol: -P 2ybc	Mo *K*α radiation, λ = 0.71073 Å
*a* = 9.9393 (5) Å	Cell parameters from 6105 reflections
*b* = 17.9047 (9) Å	θ = 1.9–29.0°
*c* = 13.2532 (7) Å	µ = 0.17 mm^−^^1^
β = 100.715 (1)°	*T* = 297 K
*V* = 2317.4 (2) Å^3^	Block, orange
*Z* = 4	0.47 × 0.28 × 0.27 mm

### Data collection

**Table:**

Bruker SMART APEXII CCD area-detector. diffractometer	6105 independent reflections
Radiation source: sealed tube	3770 reflections with *I* > 2σ(*I*)
graphite	*R*_int_ = 0.030
φ and ω scans	θ_max_ = 29.0°, θ_min_ = 1.9°
Absorption correction: multi-scan (*SADABS*; Bruker, 2009)	*h* = −13→13
*T*_min_ = 0.924, *T*_max_ = 0.955	*k* = −24→24
23078 measured reflections	*l* = −17→18

### Refinement

**Table:**

Refinement on *F*^2^	Primary atom site location: structure-invariant direct methods
Least-squares matrix: full	Secondary atom site location: difference Fourier map
*R*[*F*^2^ > 2σ(*F*^2^)] = 0.057	Hydrogen site location: inferred from neighbouring sites
*wR*(*F*^2^) = 0.173	H-atom parameters constrained
*S* = 1.04	*w* = 1/[σ^2^(*F*_o_^2^) + (0.0718*P*)^2^ + 0.6034*P*] where *P* = (*F*_o_^2^ + 2*F*_c_^2^)/3
6105 reflections	(Δ/σ)_max_ = 0.001
293 parameters	Δρ_max_ = 0.37 e Å^−^^3^
0 restraints	Δρ_min_ = −0.34 e Å^−^^3^

### Special details

**Table:**

Geometry. All e.s.d.'s (except the e.s.d. in the dihedral angle between two l.s. planes) are estimated using the full covariance matrix. The cell e.s.d.'s are taken into account individually in the estimation of e.s.d.'s in distances, angles and torsion angles; correlations between e.s.d.'s in cell parameters are only used when they are defined by crystal symmetry. An approximate (isotropic) treatment of cell e.s.d.'s is used for estimating e.s.d.'s involving l.s. planes.
Refinement. Refinement of *F*^2^ against ALL reflections. The weighted *R*-factor *wR* and goodness of fit *S* are based on *F*^2^, conventional *R*-factors *R* are based on *F*, with *F* set to zero for negative *F*^2^. The threshold expression of *F*^2^ > σ(*F*^2^) is used only for calculating *R*-factors(gt) *etc*. and is not relevant to the choice of reflections for refinement. *R*-factors based on *F*^2^ are statistically about twice as large as those based on *F*, and *R*- factors based on ALL data will be even larger.

### Fractional atomic coordinates and isotropic or equivalent isotropic displacement parameters (Å^2^)

**Table:**

	*x*	*y*	*z*	*U*_iso_*/*U*_eq_	Occ. (<1)
N1	0.37923 (17)	0.19719 (9)	0.60635 (12)	0.0544 (4)	
N2	0.5404 (2)	−0.11253 (14)	0.11672 (19)	0.0943 (7)	
C1	0.3917 (3)	0.24752 (13)	0.68435 (19)	0.0749 (7)	
H1A	0.3134	0.2636	0.7070	0.090*	
C2	0.5133 (3)	0.27431 (15)	0.7291 (2)	0.0861 (8)	
H2A	0.5193	0.3085	0.7826	0.103*	
C3	0.6310 (3)	0.25112 (14)	0.69564 (19)	0.0779 (7)	
H3A	0.7163	0.2697	0.7260	0.093*	
C4	0.6188 (2)	0.20033 (12)	0.61701 (16)	0.0601 (5)	
H4A	0.6970	0.1843	0.5943	0.072*	
C5	0.49121 (19)	0.17222 (10)	0.57031 (14)	0.0466 (4)	
C6	0.47237 (18)	0.12106 (10)	0.48520 (14)	0.0478 (4)	
H6A	0.3837	0.1055	0.4583	0.057*	
C7	0.57404 (18)	0.09435 (10)	0.44222 (14)	0.0470 (4)	
H7A	0.6621	0.1100	0.4708	0.056*	
C8	0.56174 (17)	0.04385 (9)	0.35634 (14)	0.0447 (4)	
C9	0.67804 (19)	0.02127 (12)	0.31950 (16)	0.0602 (5)	
H9A	0.7627	0.0407	0.3498	0.072*	
C10	0.6720 (2)	−0.02858 (13)	0.24028 (17)	0.0677 (6)	
H10A	0.7524	−0.0421	0.2185	0.081*	
C11	0.5480 (2)	−0.05947 (12)	0.19157 (16)	0.0602 (5)	
C12	0.4306 (2)	−0.03423 (12)	0.22563 (17)	0.0630 (5)	
H12A	0.3453	−0.0516	0.1932	0.076*	
C13	0.43791 (19)	0.01503 (11)	0.30494 (16)	0.0568 (5)	
H13A	0.3574	0.0298	0.3253	0.068*	
C14	0.4085 (3)	−0.14535 (16)	0.0674 (2)	0.0988 (10)	
H14A	0.4247	−0.1945	0.0412	0.119*	
H14B	0.3509	−0.1514	0.1184	0.119*	
C15	0.3368 (4)	−0.09968 (19)	−0.0164 (3)	0.1244 (12)	
H15A	0.2533	−0.1241	−0.0475	0.187*	
H15B	0.3938	−0.0929	−0.0667	0.187*	
H15C	0.3159	−0.0519	0.0098	0.187*	
C16A	0.6609 (5)	−0.1534 (2)	0.0964 (3)	0.0785 (14)	0.738 (9)
H16A	0.6350	−0.2045	0.0777	0.094*	0.738 (9)
H16B	0.7302	−0.1546	0.1585	0.094*	0.738 (9)
C17A	0.7198 (5)	−0.1177 (2)	0.0113 (4)	0.0975 (17)	0.738 (9)
H17A	0.7939	−0.1477	−0.0033	0.146*	0.738 (9)
H17B	0.7530	−0.0687	0.0321	0.146*	0.738 (9)
H17C	0.6499	−0.1140	−0.0492	0.146*	0.738 (9)
C16B	0.6536 (13)	−0.1042 (7)	0.0426 (10)	0.080 (4)*	0.262 (9)
H16C	0.6142	−0.1116	−0.0293	0.096*	0.262 (9)
H16D	0.7013	−0.0567	0.0518	0.096*	0.262 (9)
C17B	0.7432 (15)	−0.1684 (7)	0.0869 (10)	0.090 (4)*	0.262 (9)
H17D	0.8044	−0.1811	0.0414	0.135*	0.262 (9)
H17E	0.6870	−0.2108	0.0949	0.135*	0.262 (9)
H17F	0.7953	−0.1544	0.1526	0.135*	0.262 (9)
S1	0.04707 (5)	0.15726 (4)	0.24744 (4)	0.0654 (2)	
O1	0.0265 (2)	0.18007 (11)	0.14137 (14)	0.0980 (6)	
O2	−0.0623 (2)	0.18000 (11)	0.29822 (16)	0.0985 (6)	
O3	0.18028 (17)	0.17776 (11)	0.30344 (15)	0.0916 (6)	
C19	0.0540 (2)	0.02197 (16)	0.3415 (2)	0.0776 (7)	
H19A	0.0560	0.0495	0.4013	0.093*	
C20	0.0584 (3)	−0.0546 (2)	0.3458 (3)	0.1078 (11)	
H20A	0.0638	−0.0787	0.4085	0.129*	
C21	0.0549 (3)	−0.0958 (2)	0.2577 (5)	0.1239 (16)	
H21A	0.0578	−0.1477	0.2608	0.149*	
C22	0.0472 (3)	−0.0597 (2)	0.1643 (3)	0.1092 (11)	
H22A	0.0446	−0.0874	0.1047	0.131*	
C23	0.0431 (2)	0.01794 (17)	0.1597 (2)	0.0815 (7)	
H23A	0.0381	0.0423	0.0972	0.098*	
C24	0.04664 (18)	0.05846 (14)	0.24854 (17)	0.0618 (5)	
C18	0.2404 (2)	0.17099 (14)	0.56214 (19)	0.0718 (6)	
H18A	0.2365	0.1176	0.5682	0.108*	
H18B	0.2185	0.1848	0.4910	0.108*	
H18C	0.1757	0.1934	0.5984	0.108*	
O1W	0.8847 (2)	0.20924 (15)	0.49628 (17)	0.1225 (8)	
H1W1	0.9246	0.2080	0.4349	0.147*	
H2W1	0.9407	0.2522	0.5417	0.147*	

### Atomic displacement parameters (Å^2^)

**Table:**

	*U*^11^	*U*^22^	*U*^33^	*U*^12^	*U*^13^	*U*^23^
N1	0.0629 (10)	0.0548 (9)	0.0489 (9)	0.0076 (7)	0.0193 (8)	0.0020 (7)
N2	0.0730 (13)	0.1111 (17)	0.0977 (17)	0.0068 (12)	0.0129 (12)	−0.0540 (14)
C1	0.1001 (19)	0.0667 (14)	0.0643 (14)	0.0145 (13)	0.0324 (14)	−0.0063 (12)
C2	0.126 (2)	0.0707 (15)	0.0626 (15)	−0.0013 (16)	0.0201 (16)	−0.0207 (13)
C3	0.0946 (18)	0.0713 (15)	0.0622 (14)	−0.0176 (13)	0.0001 (13)	−0.0076 (12)
C4	0.0608 (12)	0.0635 (12)	0.0542 (12)	−0.0035 (9)	0.0060 (9)	−0.0027 (10)
C5	0.0511 (10)	0.0446 (9)	0.0452 (10)	0.0039 (7)	0.0121 (8)	0.0037 (8)
C6	0.0438 (9)	0.0507 (10)	0.0494 (10)	−0.0008 (7)	0.0096 (8)	−0.0008 (8)
C7	0.0417 (9)	0.0503 (10)	0.0479 (10)	0.0036 (7)	0.0053 (7)	0.0022 (8)
C8	0.0415 (9)	0.0446 (9)	0.0478 (10)	0.0047 (7)	0.0082 (7)	0.0028 (8)
C9	0.0391 (9)	0.0771 (13)	0.0641 (13)	0.0035 (9)	0.0083 (9)	−0.0135 (11)
C10	0.0471 (11)	0.0884 (15)	0.0693 (14)	0.0122 (10)	0.0149 (10)	−0.0182 (12)
C11	0.0587 (12)	0.0614 (12)	0.0602 (12)	0.0083 (9)	0.0099 (10)	−0.0118 (10)
C12	0.0474 (11)	0.0695 (13)	0.0704 (14)	−0.0030 (9)	0.0066 (9)	−0.0172 (11)
C13	0.0429 (10)	0.0630 (12)	0.0660 (13)	0.0021 (8)	0.0139 (9)	−0.0100 (10)
C14	0.116 (2)	0.0828 (18)	0.088 (2)	0.0180 (16)	−0.0052 (17)	−0.0326 (16)
C15	0.157 (3)	0.102 (2)	0.104 (3)	0.030 (2)	−0.003 (2)	−0.008 (2)
C16A	0.083 (3)	0.065 (2)	0.091 (3)	0.0100 (17)	0.027 (2)	−0.0236 (19)
C17A	0.106 (3)	0.096 (3)	0.100 (3)	−0.007 (2)	0.042 (3)	−0.022 (2)
S1	0.0521 (3)	0.0842 (4)	0.0600 (4)	−0.0066 (3)	0.0107 (2)	0.0051 (3)
O1	0.1207 (16)	0.1058 (14)	0.0630 (11)	0.0042 (12)	0.0051 (10)	0.0237 (10)
O2	0.0845 (13)	0.1046 (14)	0.1157 (16)	0.0043 (10)	0.0427 (12)	−0.0098 (12)
O3	0.0688 (11)	0.1090 (14)	0.0908 (13)	−0.0309 (10)	−0.0008 (9)	0.0101 (11)
C19	0.0459 (11)	0.1011 (19)	0.0840 (17)	−0.0052 (11)	0.0073 (11)	0.0224 (15)
C20	0.0568 (16)	0.111 (3)	0.151 (3)	−0.0014 (16)	0.0091 (18)	0.052 (2)
C21	0.0538 (16)	0.085 (2)	0.229 (5)	0.0030 (14)	0.016 (2)	0.023 (3)
C22	0.0698 (18)	0.098 (2)	0.161 (3)	0.0011 (16)	0.022 (2)	−0.028 (2)
C23	0.0588 (14)	0.100 (2)	0.0860 (18)	−0.0017 (13)	0.0138 (12)	−0.0064 (15)
C24	0.0338 (9)	0.0847 (15)	0.0659 (13)	−0.0028 (9)	0.0066 (8)	0.0094 (12)
C18	0.0523 (12)	0.0903 (17)	0.0775 (16)	0.0081 (11)	0.0242 (11)	0.0007 (13)
O1W	0.1150 (17)	0.157 (2)	0.1059 (16)	−0.0526 (15)	0.0468 (13)	−0.0250 (15)

### Geometric parameters (Å, °)

**Table:**

N1—C1	1.360 (3)	C15—H15B	0.9600
N1—C5	1.365 (2)	C15—H15C	0.9600
N1—C18	1.472 (3)	C16A—C17A	1.506 (7)
N2—C11	1.365 (3)	C16A—H16A	0.9700
N2—C16A	1.471 (4)	C16A—H16B	0.9700
N2—C14	1.474 (4)	C17A—H17A	0.9600
N2—C16B	1.632 (14)	C17A—H17B	0.9600
C1—C2	1.333 (4)	C17A—H17C	0.9600
C1—H1A	0.9300	C16B—C17B	1.51 (2)
C2—C3	1.389 (4)	C16B—H16C	0.9700
C2—H2A	0.9300	C16B—H16D	0.9700
C3—C4	1.371 (3)	C17B—H17D	0.9600
C3—H3A	0.9300	C17B—H17E	0.9600
C4—C5	1.397 (3)	C17B—H17F	0.9600
C4—H4A	0.9300	S1—O2	1.4395 (18)
C5—C6	1.438 (3)	S1—O3	1.4398 (17)
C6—C7	1.337 (2)	S1—O1	1.4414 (18)
C6—H6A	0.9300	S1—C24	1.769 (2)
C7—C8	1.441 (2)	C19—C20	1.372 (4)
C7—H7A	0.9300	C19—C24	1.384 (3)
C8—C13	1.390 (3)	C19—H19A	0.9300
C8—C9	1.396 (2)	C20—C21	1.376 (5)
C9—C10	1.371 (3)	C20—H20A	0.9300
C9—H9A	0.9300	C21—C22	1.385 (5)
C10—C11	1.396 (3)	C21—H21A	0.9300
C10—H10A	0.9300	C22—C23	1.391 (4)
C11—C12	1.401 (3)	C22—H22A	0.9300
C12—C13	1.364 (3)	C23—C24	1.377 (3)
C12—H12A	0.9300	C23—H23A	0.9300
C13—H13A	0.9300	C18—H18A	0.9600
C14—C15	1.454 (4)	C18—H18B	0.9600
C14—H14A	0.9700	C18—H18C	0.9600
C14—H14B	0.9700	O1W—H1W1	0.9693
C15—H15A	0.9600	O1W—H2W1	1.0669
			
C1—N1—C5	121.22 (19)	C14—C15—H15B	109.5
C1—N1—C18	117.38 (19)	H15A—C15—H15B	109.5
C5—N1—C18	121.40 (17)	C14—C15—H15C	109.5
C11—N2—C16A	122.8 (2)	H15A—C15—H15C	109.5
C11—N2—C14	121.6 (2)	H15B—C15—H15C	109.5
C16A—N2—C14	114.1 (2)	N2—C16A—C17A	111.7 (4)
C11—N2—C16B	115.0 (5)	N2—C16A—H16A	109.3
C14—N2—C16B	115.2 (5)	C17A—C16A—H16A	109.3
C2—C1—N1	121.5 (2)	N2—C16A—H16B	109.3
C2—C1—H1A	119.2	C17A—C16A—H16B	109.3
N1—C1—H1A	119.2	H16A—C16A—H16B	108.0
C1—C2—C3	119.9 (2)	C17B—C16B—N2	96.9 (10)
C1—C2—H2A	120.0	C17B—C16B—H16C	112.4
C3—C2—H2A	120.0	N2—C16B—H16C	112.4
C4—C3—C2	118.7 (2)	C17B—C16B—H16D	112.4
C4—C3—H3A	120.7	N2—C16B—H16D	112.4
C2—C3—H3A	120.7	H16C—C16B—H16D	109.9
C3—C4—C5	121.3 (2)	C16B—C17B—H17D	109.5
C3—C4—H4A	119.3	C16B—C17B—H17E	109.5
C5—C4—H4A	119.3	H17D—C17B—H17E	109.5
N1—C5—C4	117.31 (17)	C16B—C17B—H17F	109.5
N1—C5—C6	119.16 (17)	H17D—C17B—H17F	109.5
C4—C5—C6	123.50 (17)	H17E—C17B—H17F	109.5
C7—C6—C5	124.29 (17)	O2—S1—O3	112.89 (13)
C7—C6—H6A	117.9	O2—S1—O1	113.17 (13)
C5—C6—H6A	117.9	O3—S1—O1	112.36 (12)
C6—C7—C8	126.94 (17)	O2—S1—C24	106.01 (11)
C6—C7—H7A	116.5	O3—S1—C24	104.71 (11)
C8—C7—H7A	116.5	O1—S1—C24	106.92 (12)
C13—C8—C9	115.87 (17)	C20—C19—C24	120.3 (3)
C13—C8—C7	123.87 (16)	C20—C19—H19A	119.9
C9—C8—C7	120.26 (16)	C24—C19—H19A	119.9
C10—C9—C8	122.39 (18)	C19—C20—C21	120.3 (3)
C10—C9—H9A	118.8	C19—C20—H20A	119.8
C8—C9—H9A	118.8	C21—C20—H20A	119.8
C9—C10—C11	121.40 (18)	C20—C21—C22	119.7 (4)
C9—C10—H10A	119.3	C20—C21—H21A	120.1
C11—C10—H10A	119.3	C22—C21—H21A	120.1
N2—C11—C10	122.44 (19)	C21—C22—C23	120.1 (4)
N2—C11—C12	121.4 (2)	C21—C22—H22A	119.9
C10—C11—C12	116.11 (19)	C23—C22—H22A	119.9
C13—C12—C11	121.91 (19)	C24—C23—C22	119.5 (3)
C13—C12—H12A	119.0	C24—C23—H23A	120.2
C11—C12—H12A	119.0	C22—C23—H23A	120.2
C12—C13—C8	122.21 (17)	C23—C24—C19	120.0 (3)
C12—C13—H13A	118.9	C23—C24—S1	121.29 (19)
C8—C13—H13A	118.9	C19—C24—S1	118.6 (2)
C15—C14—N2	112.6 (3)	N1—C18—H18A	109.5
C15—C14—H14A	109.1	N1—C18—H18B	109.5
N2—C14—H14A	109.1	H18A—C18—H18B	109.5
C15—C14—H14B	109.1	N1—C18—H18C	109.5
N2—C14—H14B	109.1	H18A—C18—H18C	109.5
H14A—C14—H14B	107.8	H18B—C18—H18C	109.5
C14—C15—H15A	109.5	H1W1—O1W—H2W1	103.8
			
C5—N1—C1—C2	−0.3 (3)	C10—C11—C12—C13	−2.9 (4)
C18—N1—C1—C2	180.0 (2)	C11—C12—C13—C8	0.6 (4)
N1—C1—C2—C3	0.4 (4)	C9—C8—C13—C12	2.1 (3)
C1—C2—C3—C4	−0.4 (4)	C7—C8—C13—C12	−178.2 (2)
C2—C3—C4—C5	0.3 (3)	C11—N2—C14—C15	83.8 (4)
C1—N1—C5—C4	0.2 (3)	C16A—N2—C14—C15	−109.7 (3)
C18—N1—C5—C4	179.92 (18)	C16B—N2—C14—C15	−63.0 (6)
C1—N1—C5—C6	−177.75 (18)	C11—N2—C16A—C17A	−95.3 (4)
C18—N1—C5—C6	2.0 (3)	C14—N2—C16A—C17A	98.4 (4)
C3—C4—C5—N1	−0.2 (3)	C16B—N2—C16A—C17A	−3.2 (7)
C3—C4—C5—C6	177.6 (2)	C11—N2—C16B—C17B	106.1 (7)
N1—C5—C6—C7	178.44 (17)	C16A—N2—C16B—C17B	−6.1 (5)
C4—C5—C6—C7	0.7 (3)	C14—N2—C16B—C17B	−104.8 (7)
C5—C6—C7—C8	−179.19 (17)	C24—C19—C20—C21	−0.3 (4)
C6—C7—C8—C13	−0.1 (3)	C19—C20—C21—C22	0.1 (4)
C6—C7—C8—C9	179.54 (19)	C20—C21—C22—C23	0.1 (4)
C13—C8—C9—C10	−2.6 (3)	C21—C22—C23—C24	−0.1 (4)
C7—C8—C9—C10	177.8 (2)	C22—C23—C24—C19	0.0 (3)
C8—C9—C10—C11	0.2 (4)	C22—C23—C24—S1	178.04 (19)
C16A—N2—C11—C10	13.7 (4)	C20—C19—C24—C23	0.3 (3)
C14—N2—C11—C10	179.0 (3)	C20—C19—C24—S1	−177.88 (18)
C16B—N2—C11—C10	−34.1 (6)	O2—S1—C24—C23	127.83 (19)
C16A—N2—C11—C12	−164.7 (3)	O3—S1—C24—C23	−112.59 (18)
C14—N2—C11—C12	0.6 (4)	O1—S1—C24—C23	6.8 (2)
C16B—N2—C11—C12	147.5 (5)	O2—S1—C24—C19	−54.06 (19)
C9—C10—C11—N2	−176.0 (2)	O3—S1—C24—C19	65.52 (19)
C9—C10—C11—C12	2.5 (4)	O1—S1—C24—C19	−175.08 (17)
N2—C11—C12—C13	175.6 (2)		

### Hydrogen-bond geometry (Å, °)

**Table:**

*Cg*1 is the centroid of the C19–C24 ring.

**Table:**

*D*—H···*A*	*D*—H	H···*A*	*D*···*A*	*D*—H···*A*
O1W—H1W1···O2^i^	0.97	1.91	2.821 (3)	156
O1W—H2W1···O1^ii^	1.07	1.88	2.933 (3)	170
C1—H1A···O3^iii^	0.93	2.26	3.151 (3)	160
C3—H3A···O2^ii^	0.93	2.41	3.335 (4)	178
C4—H4A···O1W	0.93	2.50	3.338 (3)	149
C18—H18B···O3	0.96	2.45	3.371 (3)	162
C10—H10A···Cg1^i^	0.93	2.95	3.741 (2)	144

Symmetry codes: (i) *x*+1, *y*, *z*; (ii) *x*+1, −*y*+1/2, *z*+1/2; (iii) *x*, −*y*+1/2, *z*+1/2.
